# A Case Report and Literature Review on Positivity for Multiple Antibodies: Autoimmune Encephalitis in Focus

**DOI:** 10.7759/cureus.50393

**Published:** 2023-12-12

**Authors:** Saad Asbeutah, Kamel Alhashime, Maryam Alhamer

**Affiliations:** 1 Neurology, Kuwait University, Kuwait City, KWT; 2 Internal Medicine, Kuwait Board of Internal Medicine, Kuwait City, KWT

**Keywords:** immunotherapy, neuroimmunology, neuropsychiatric conditions, neuronal surface antibodies, autoimmune encephalitis

## Abstract

Autoimmune encephalitis (AE) is a group of neuropsychiatric disorders caused by antibodies that target the neuronal surface, synaptic, or intracellular antigens, impairing brain function. Although AE can affect people of different age groups, the occurrence of AE within specific age brackets depends on the specific type of AE and the antibodies produced. While AE is frequently considered a paraneoplastic syndrome linked to cancer, it is essential to acknowledge that the intensity of this association can vary depending on the specific antibody, leading to diverse relationships with paraneoplastic syndromes. Numerous cases have been recorded where AE manifests without an underlying malignancy. The diagnostic criteria for AE are characterized by a subacute deterioration of cognition, altered mental status, or psychiatric symptoms. Immunotherapy is recommended as a treatment for the condition, and the prognosis varies depending on the subtype. In this case, we present the case of an elderly woman who showed acute mental status changes, psychiatric symptoms, EEG alterations, and positive antibody results in both serum and CSF. Our case breaks new ground as the first documented instance of a female with positive serum anti-LGI 1, anti-AMPAR2, anti-Ri, and anti-CENP-A/B antibodies.

## Introduction

Autoimmune encephalitis (AE) is a term used to describe a group of immune-related neuropsychiatric conditions that are often linked to antibodies. These antibodies affect the neuronal surface, synaptic, or intracellular antigens, impairing brain function. Several antibodies are associated with AE, each presenting with a different clinical pattern and characteristic symptoms [1]. 

While AE can impact individuals across various age groups, the prevalence of AE within specific age ranges is contingent upon the particular type of AE and the antibodies generated. For instance, NMDA receptor encephalitis is typically observed in children and young adults while LGI1 encephalitis is more prevalent among older men [2]. While AE is often perceived as a paraneoplastic syndrome linked to cancer, it is essential to note that the degree of association may vary depending on the specific antibody involved, encompassing a range of relationships with paraneoplastic syndromes. Multiple instances have been documented where AE occurs independently of an underlying malignancy [3].

Over the past decade, research has led to new syndromes and biomarkers, transforming the diagnostic approaches for these disorders. Present diagnostic criteria for autoimmune limbic encephalitis require a subacute onset of short-term memory loss, seizures, or psychiatric symptoms. These symptoms should be accompanied by bilateral abnormalities in brain MRI in the temporal lobes, cerebrospinal fluid (CSF) pleocytosis, or EEG displaying slow wave activity or epileptic changes. If any of these symptoms are absent, the diagnosis of AE relies on the crucial detection of antibodies against cell-surface or intracellular neuronal proteins [4].

Treatment for AE involves intensive immunotherapy, typically involving a combination of corticosteroids, intravenous immunoglobulin, plasma exchange, and, in some cases, anti-CD20 therapy. Alternative treatments are also available, and the prognosis varies depending on the subtype of AE and the presence of underlying cancer [5].

We present a case involving an elderly female who exhibited acute shifts in mental status, psychiatric symptoms, distinctive EEG changes, and positive results for multiple antibodies in both serum and CSF, which are all indicative of AE.

## Case presentation

A 62-year-old woman with a medical history including type 2 diabetes mellitus and dyslipidemia was brought to the emergency department by her family due to noticeable changes in her mental state. Previously, she had been independent and capable of performing daily activities. However, with a subacute onset of symptoms, she began experiencing difficulties in recalling recent events and mild disorientation. She also suffered from persecutory delusions and visual hallucinations, described as seeing unfamiliar people in the room. These symptoms were not accompanied by aggression or disturbed sleep. She did not exhibit fever, headache, joint pain, or other constitutional symptoms. There was no prior history of psychiatric conditions or autoimmune disorders, and her family lacked a history of autoimmune issues, malignancy, or dementia. Over time, her memory loss intensified, and her functional abilities declined to the point where she required assistance with walking, eating, and using a Foley catheter for urination.

In the emergency department, her vital signs included: heart rate 83/minute, blood pressure 122/77, respiratory rate 18/minute, temperature 99.6 F, and blood glucose 145 mg/dl. During the physical examination, the woman appeared alert but lacked orientation to time, place, and person. Although awake, her communication was restricted to following simple commands, and she struggled with more intricate sentence structures. The patient was moving all four limbs; upper limbs were 5/5 and lower limbs were 4/5 on the Medical Research Council (MRC) scale. There were absent lower limb reflexes and urinary incontinence.

Apart from an elevated C-reactive protein, the complete blood count and comprehensive metabolic panel showed no noteworthy abnormalities. Following the initiation of empiric meningitis antibiotics, a lumbar puncture was performed. The cerebrospinal fluid (CSF) analysis exhibited fewer than five red blood cells per cubic millimeter, fewer than five white blood cells per cubic millimeter, a protein level of 819 mg/dL, and a glucose level of 205.2 mg/dL. Gram staining demonstrated a minimal presence of pus cells, and no organisms were cultured after a four-day incubation period, prompting the discontinuation of antibiotics. A polymerase chain reaction (PCR) on the CSF did not detect cytomegalovirus (CMV), Epstein-Barr virus, TB, John Cunningham (JC) virus, Herpes simplex virus (HSV), and enterovirus microbes. Additionally, blood specimens tested negative for HIV, and urine toxicology came back clean. A routine EEG showed diffuse slow theta waves without evidence of seizure activity, concurring with encephalopathy.

**Figure 1:**
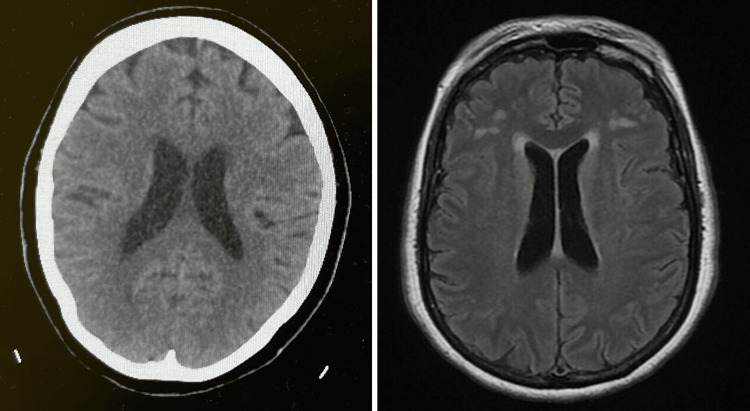
Head CT (left): Periventricular and deep white matter hypodensities, providing clinically significant indicators of chronic ischemic changes associated with small vessel disease. MRI brain scan with intravenous contrast (right): mild periventricular white matter diffusion, variable-sized lesions, hyperintense signals on FLAIR and T2W images. Findings suggest chronic ischemic infarcts and small vessel disease. Mild ventricular dilatation and widening CSF spaces are noted. FLAIR: fluid-attenuated inversion recovery; T2W: T2-weighted

Imaging studies were subsequently conducted to delve deeper into the underlying condition. A CT head illuminated periventricular and deep white matter hypodensities, indicative of chronic ischemic changes associated with small vessel disease. In-depth insights were gained through MRI brain scans with intravenous contrast, revealing mild diffuse periventricular white matter and an array of variable-sized abnormal signal intensity lesions distributed throughout both frontoparietal, periventricular, and right posterior parietal subcortical white matter. These lesions manifested hyperintense signals on fluid-attenuated inversion recovery (FLAIR) and T2-weighted (T2W) images while presenting iso-hypodense signals on T1W images. Notably, there was no restriction on DWI, and no perilesional edema or mass effect was observed. The absence of significant blooming in susceptibility-weighted angiography (SWAN) further underscored the diagnostic observations. These findings align with chronic ischemic infarcts in the white matter and characteristic changes associated with small vessel disease. Additionally, mild dilatation of the ventricular systems and widening cerebrospinal fluid spaces along the basal cistern and cortical sulci were noted, indicative of brain involutional changes. Both head CT and MRI brain scans are displayed in Figure 1.

**Figure 2:**
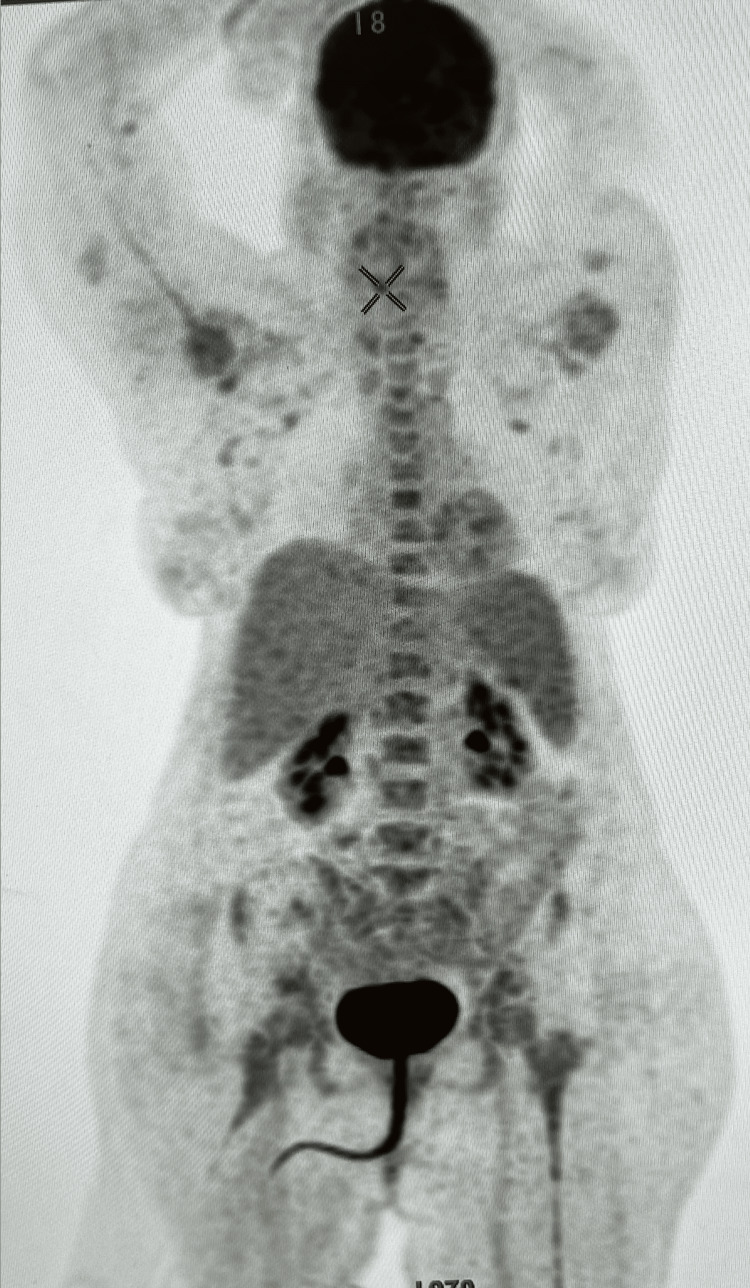
A thorough nuclear medicine PET/CT scan unveils moderate uptake patterns in the bone marrow and spleen, potentially signifying an underlying autoimmune state, nearly excluding underlying malignancy, all-encompassing from vertex to mid-thigh PET: positron emission tomography

In light of inconclusive results from the infectious investigation, attention turned toward a heightened consideration of paraneoplastic syndrome or AE. Specific antibodies targeting autoimmune and paraneoplastic encephalitis were promptly dispatched. Meanwhile, as the medical team awaited results, high-dose pulse corticosteroids were initiated (1 g/day for five days). The serum autoimmune panel for encephalitis later revealed positive findings for anti-LGI 1 (1/100), anti-AMPAR2 (1/1000), anti-Ri, and weak anti-CENP-A/B. Remarkably, the former two antibodies also tested positive in the cerebrospinal fluid (CSF) samples. A comprehensive nuclear medicine positron emission tomography (PET)/CT scan, spanning from the vertex to mid-thigh, depicted moderate uptake in the bone marrow and spleen, indicating a potential autoimmune condition and less likely of hematological origin (Figure 2).

After completing a five-day pulse corticosteroid course, the patient underwent a five-day intravenous immunoglobulin (IVIG) therapy regimen (400 mg/kg/day for 5 days). However, notable symptom improvements were only observed on the fifth day of treatment. Following that, the patient underwent rituximab therapy twice (1 g IV infusion, spaced 15 days apart) as the subsequent step in the therapeutic regimen. Remarkably, the patient exhibited substantial improvement post-rituximab therapy, displaying heightened engagement, accurate responses to questions, improved orientation, and a notable absence of errors in identifying family members and friends.

## Discussion

Diagnosing AE can be intricate, especially in this patient's age group, where it may not be a commonly anticipated differential. During the initial visit to the emergency department, infections such as infectious meningitis and encephalitis were more prominently considered due to their higher likelihood. The possibility of AE was initially deemed lower, as there was no history of autoimmune diseases, and minimal concern for cancer emerged during the diagnostic workup.

A thorough evaluation of clinical, laboratory, and imaging findings is crucial in determining the potential conditions for differential diagnosis of autoimmune limbic encephalitis. According to Derry et al., during the initial examination, infectious causes, such as viral encephalitis and herpes simplex virus (HSV) encephalitis, are commonly considered due to their prevalence and similar presentation characteristics. Non-infectious origins like metabolic disturbances and toxic encephalopathies also require consideration. Moreover, it is essential to investigate neoplastic disorders carefully, particularly paraneoplastic limbic encephalitis associated with underlying tumors, as the varied clinical manifestations of this condition can sometimes mimic other diseases. The differential diagnosis also encompasses autoimmune diseases beyond limbic encephalitis, including systemic lupus erythematosus (SLE) and rheumatoid arthritis, which can occasionally present with neurological symptoms. A comprehensive list of differential diagnoses adapted from Derry et al. is listed in Table 1 [6].

**Table 1:** A thorough and exhaustive compilation of potential alternative diagnoses Adapted from Derry et al. [6]

Classification	Etiology
Metabolic/toxic encephalopathy	Drugs, hypo/hyperglycemia, electrolyte disturbances
Psychiatric disorders	Schizophreniform psychosis, major depressive disorder, bipolar disorder
Infection	HSV encephalitis, enterovirus encephalitis
Inflammation	Hashimoto's encephalopathy
Malignancy	Primary brain cancer, metastatic brain cancer
Neurodegenerative disorders	Alzheimer's disease, vascular dementia, Parkinson's disease

Various antibodies have been identified in association with AE, each of which can result in different symptoms and outcomes. Among the most significant antibodies linked to this condition are anti-N-methyl-D-aspartate receptor (NMDAR) antibodies, anti-leucine-rich glioma-inactivated 1 (LGI1) antibodies, and anti-contactin-associated protein-like 2 (CASPR2) antibodies. People who suffer from anti-LGI1 encephalitis may face significant difficulties in visuospatial and executive functions, while anti-NMDAR encephalitis is often associated with language deficits and behavioral changes [7]. In the specific case we are discussing, the patient had anti-Ri, anti-CENP-A/B, anti-LGI 1, and anti-AMPAR2 in her serum and exhibited a picture more consistent with limbic encephalitis. The neurocognitive symptoms in our patient, together with markedly elevated protein levels in the cerebrospinal fluid, and peripheral blood markers, such as C-reactive protein, along with the presence of specific autoantibodies, collectively indicate neuroinflammation. This combination of clinical, laboratory, and immunological evidence underscores the inflammatory processes affecting the nervous system.

Ongoing research is helping identify new antibodies and their role in AE. A comprehensive table of these antibodies and their features adapted from multiple articles is provided in Table 2 [8-11].

**Table 2:** Comprehensive list detailing various antibodies and their specific roles in the context of autoimmune and paraneoplastic encephalitis Adapted from articles [8-11] FDG: fluorodeoxyglucose; PET: positron emission tomography

Antibody	Clinical presentation	Brain imaging findings	Frequency of cancer (%)	Usual tumors
DPPX	Prodromal weight loss or diarrhea, encephalopathy and CNS hyperexcitability	Normal or non-specific changes on brain MRI	N/A	Lymphoma
GABAa receptor	Seizures, rapidly progressive encephalopathy, movement disorders	Brain MRI shows T2/FLAIR hyperintensities which span an extensive range of cortical and subcortical structures, often show asynchronously evolution, and are potentially treatment-responsive	30	Thymoma
GABAb receptor	Seizures (100%), cognitive, behavioral, and psychiatric deficits	Brain MRI invariably consists of unilateral or bilateral mesiotemporal T2/FLAIR hyperintensity, with additional less commonly affected areas including the corpus callosum and brainstem	50	Small cell lung cancer
IgLON5	Sleep disorders, bulbar syndrome, gait instability, cognitive impairment, and movement disorders	Brain atrophy, T2 hyperintensity, and white matter changes have all been observed on brain MRI, in addition to other signs mainly involving the hippocampus, brainstem, white matter, cerebellum, and cortex. High-convexity tight sulci (defined as the compression of sulci at the vertex, enlarged CSF spaces in the Sylvian fissure, and ventriculomegaly) on MRI, a marker of CSF dynamics problems, has been demonstrated in one patient	N/A	N/A
GQ1b	Ptosis, ophthalmoplegia (mostly complete), facial palsy, limb ataxia, dysarthria, and areflexia	High-intensity areas on T2-weighted images of the brainstem, thalamus, cerebellum, and cerebrum (30% of patients)	N/A	N/A
Ri (ANNA-2)	Brainstem/cerebellar syndrome, opsoclonus-myoclonus-ataxia syndrome	N/A	>70	Small cell and Non-small cell lung cancer, breast cancer
AMPAR	Limbic encephalitis	FDG-PET abnormalities include hippocampal hypermetabolism, widespread cortical hypometabolism, and cerebellar hypermetabolism	>50	Small cell lung cancer
LGI1	Limbic encephalitis	N/A	<10	Malignant thymoma, neuroendocrine tumors
Hu (ANNA-1)	Sensory neuronopathy, chronic gastrointestinal pseudo-obstruction, encephalomyelitis	N/A	85	Small cell and Non-small cell lung cancer
NMDAR	Confusion, seizures, and reduced conscious state	N/A	38	Ovarian teratoma
Caspr2	Morvan syndrome, limbic encephalitis, acquired neuromyotonia	N/A	50 ( Morvan syndrome)	Malignant thymoma
AQP4	Neuromyelitis optica spectrum disorder	N/A	<5	Adenocarcinomas
mGluR5	Cerebellar ataxia	N/A	30	Mostly hematologic
PCA-2 (MAP1B)	Sensorimotor neuropathy, rapidly progressive cerebellar syndrome, encephalomyelitis	N/A	80	Small cell and Non-small cell lung cancer, breast cancer

The co-occurrence of multiple auto-antibodies is a rare phenomenon, as evidenced by Ren Haitao's extensive study involving 531 cases of AE, where merely 10 cases demonstrated the presence of multiple anti-neuronal antibodies. Our case is the first documented instance of a female with positive serum anti-LGI 1, anti-AMPAR2, anti-Ri, and weak anti-CENP-A/B antibodies. Ren Haitao's findings underscore the exceptional nature of such coexistence, citing instances like anti-GABABR/anti-Hu and anti-NMDAR/APQ-4. Our case, presenting these four antibodies simultaneously, stands out as a unique and unparalleled occurrence in the literature [12].

In another instance of AE featuring multiple auto-antibodies, Jianhua Yang et al. present a compelling case involving a 60-year-old man exhibiting delayed response, behavioral changes, psychosis, and sleep disturbances. Diagnostic investigations revealed serum hyponatremia and positive serum LGI1 and GABABR1 antibodies. Notably, further scrutiny uncovered the presence of HHV-7 in the cerebrospinal fluid, and subsequent weeks showed an ovarian teratoma in the patient. The pathology report detailed a mature cystic teratoma in the right ovary, comprising choroid plexus, neuropil, sebaceous glands, hair follicles, and cartilage tissue. In contrast, our case, despite an extensive viral screening and thorough malignancy evaluation, showed negative results for viral agents and no evidence of cancer. This juxtaposition highlights the diverse etiologies within AE and underscores the importance of individualized diagnostic approaches [13].

Continuing the narrative, Jing Yang et al. presented a case involving a 56-year-old man who sought medical attention for cognitive decline and abnormal psychological behaviors lasting over 20 days. The subsequent diagnosis identified anti-AMPAR encephalitis, with a CT scan revealing pulmonary bullae in the upper lobe of both lungs and concurrent emphysema. Notably, four months later, the patient received an additional diagnosis of small-cell lung cancer. In contrast, our current case shows no evidence of malignancy and has successfully cleared the comprehensive malignancy screening. However, ongoing surveillance is paramount to identify any potential future signs or symptoms of cancer promptly. This parallel underscores the nuanced nature of AE and the need for vigilant monitoring in similar cases [14].

Alho et al. presented a compelling case report illustrating the prevalence of predominantly psychotic symptoms in the context of anti-LGI 1 encephalitis. Their patient demonstrated disorganized behavior and speech, along with persecutory delusions and indirect signs of auditory hallucinations during the medical examination. Initially diagnosed as unspecified psychosis, further investigations revealed noteworthy findings such as right temporal epileptiform activity in the EEG, abnormal bilateral hyperintensities in the temporal lobes in the brain MRI, and a positive titer of anti-LGI 1 antibodies in both serum and CSF. These comprehensive assessments culminated in a refined diagnosis of anti-LGI 1 limbic encephalitis. In comparison, our case exhibited a similar psychotic presentation with persecutory delusions and visual hallucinations but lacked the identical EEG and MRI findings observed in their case [15].

In a study conducted by Schäfer and colleagues, a previously healthy 24-year-old man presented with double vision and drooping eyelids, which later progressed to left-sided drooping and impaired eye movements. Positive Jolly test results suggested seronegative ocular myasthenia gravis (MG). Despite the absence of MG-associated antibodies, electromyography revealed abnormalities. Three months later, the patient reported memory loss, behavioral changes, and an unsteady gait, and was readmitted with bilateral drooping eyelids, an unstable gait, and a dystonic foot posture. Brain MRI indicated leptomeningeal contrast enhancement, which suggested inflammation, vasculitis, and limbic enhancement. CSF examination showed an increased cell count, intrathecal immunoglobulin G (IgG) synthesis, and oligoclonal bands. Subsequent CSF analysis confirmed a strong positive AMPAR reaction and later NMDAR. It is important to note that the patient's case was preceded by myasthenia gravis while the case under consideration lacked any preceding neurological signs or symptoms. Additionally, their case included oligoclonal bands, which were not seen in ours, and demonstrated limbic enhancement, which was not observed in our case [16].

Li et al. detailed the case of a man manifesting neurological symptoms 15 days post-severe acute respiratory syndrome coronavirus 2 (SARS‑CoV‑2) infection. His symptoms encompassed anxiety, paroxysmal dizziness, and involuntary movements in the left limbs and mouth, alongside sleep behavior disorders and cognitive decline with a focus on short-term memory loss. Positive anti-IgLON5 and anti-LGI1 receptor antibodies in both serum and cerebrospinal fluid led to a dual diagnosis of anti-IgLON5 disease and anti-LGI1 AE. Noteworthy symptoms included sleep behavior disorder, obstructive sleep apnea, and daytime sleepiness, along with faciobrachial dystonic seizures characteristic of anti-LGI1 encephalitis. Improvement followed high-dose steroid and mycophenolate mofetil therapy. In contrast, our case, though positive for LGI1, displayed a distinct clinical profile, lacking preexisting cold symptoms and testing negative for COVID-19. Additionally, our MRI revealed normal findings, diverging from Li et al.'s case, which demonstrated high signal intensity in the left basal ganglia. These variations underscore the heterogeneity within AE presentations [17].

Kammeyer and colleagues documented the case of a 66-year-old man experiencing a two-month history of deteriorating balance, diplopia, and confusion, devoid of prior medical conditions. The neurological examination disclosed short-term memory deficits and ataxia. Subsequent MRI revealed encephalitis affecting the brainstem, basal ganglia, and mesial temporal lobes. Despite negative infectious studies in the CSF, including herpes simplex virus polymerase chain reaction, cytology, and cytometry, the CSF analysis indicated the presence of anti-NMDA-R, anti-Ma1, and anti-Ma2. Furthermore, anti-GAD65 antibodies were found positive in the serum. While initial suspicions leaned toward a paraneoplastic process due to the patient's age, malignancy screenings yielded negative results. The EEG displayed diffuse and intermixed slowing, devoid of epileptiform discharges or seizures. Parallels with our presented case include similar EEG findings characterized by diffuse and intermixed slowing, despite the presence of different antibodies. This convergence underscores the complexity of AE presentations, emphasizing the need for comprehensive evaluations and targeted interventions [18].

Li et al. presented a case study involving a previously healthy 26-year-old female who exhibited a three-day episode of irritability, limb stiffness, sleepwalking, hallucinations, and paroxysmal mania. Initially diagnosed with schizophrenia and treated with olanzapine, the patient showed no improvement after eight days. Brain MRI revealed abnormal signals in the superior colliculus. EEG exhibited diffuse slow waves. Lumbar puncture and auto-antibody testing in CSF and serum confirmed positivity for anti-NMDAR in CSF and anti-NMDAR, and mGluR5 in serum. Malignancy screening and enhanced pelvis CT revealed an enlarged pelvic mass, later identified as bilateral ovarian teratomas (mature and immature). Surgical resection, along with intravenous steroids, immunoglobulin, oral prednisone, and chemotherapy, led to significant improvement. At the three-month follow-up, the patient was stable with resolved lesions on MRI. Our case shared a similar psychiatric presentation at the beginning with hallucinations but lacked mania and sleepwalking. While our case did not exhibit the same MRI findings, we did share the presence of diffuse slow waves in EEG [19].

Qiao et al. conducted a retrospective analysis of 22 patients with multiple coexisting antibodies across various clinical centers in China. One of the patients, a 64-year-old male, showed symptoms such as memory impairment, seizures, and faciobrachial dystonic seizures. An EEG investigation revealed increased diffuse slow-wave activity and an MRI showed an ischemic focus in the right frontal lobe. Malignancy screening yielded negative results. The CSF analysis demonstrated the presence of positive anti-NMDA and anti-LGI-1 antibodies, which were also found in the serum. This led to the diagnosis of AE. Treatment with steroids and IVIG resulted in improvement, although the patient experienced a relapse at 12 months. This case shares similarities with ours, particularly in the presence of anti-LGI-1 antibodies, initial presentation with memory deficit, and common EEG findings. The shared EEG findings further emphasize the consistency in neurophysiological manifestations associated with anti-LGI-1 AE. Recognizing these commonalities can expedite the diagnostic process and facilitate the implementation of timely and targeted interventions [20].

Table 3 condenses the salient points from all previously discussed case report summaries, spotlighting the antibodies and primary symptoms in each case.

**Table 3:** Summary of primary antibodies and signs and symptoms in aforementioned AE case reports Information obtained from [13-20] AE: autoimmune encephalitis

Case	Antibodies in serum	Primary signs and symptoms
This report	LGI 1, AMPAR2, Ri, and CENP-A/B.	Short-term memory loss, persecutory delusions, and visual hallucinations
Yang et al. (2022)	LGI1 and GABAbR1	Delayed response, behavioral changes, psychosis, and sleep disturbances
Yang et al. (2021)	AMPAR	Cognitive decline and abnormal psychological behaviors
Alho et al. (2023)	LGI1	Disorganized behavior and speech, persecutory delusions, and indirect signs of auditory hallucinations
Schäfer et al. (2023)	AMPAR and NMDAR	Loss of short-term memory, behavioral changes, fatigue, depressed mood, and unsteady gait
Li et al. (2023)	LGI1 and IgLON5	Anxiety, paroxysmal dizziness, left limbs and mouth involuntary movement, sleep behavior disorders, daytime sleepiness, paramnesia, and short-term memory loss
Kammeyer et al. (2019)	GAD65	Worsening balance, diplopia, and confusion
Li et al. (2023)	NMDAR and mGluR5	Irritability, babbling, limb stiffness, sleepwalking, hallucinations, and paroxysmal mania
Yang et al. (2022)	NMDA and LGI-1	Memory impairment, seizures, and faciobrachial dystonic seizures

While this case report offers valuable insights into the diagnosis and management of probable AE, it is crucial to acknowledge its limitations. It is important to note the presence of non-specific findings in this case, including an MRI that does not conform to the classic pattern for AE. Our findings are based on a single patient, which may limit the generalizability of the conclusions to a broader population. Furthermore, the absence of a control group hinders our ability to compare outcomes and assess the true impact of the interventions implemented. Nevertheless, this case underscores the complexity of diagnosing and managing AE, shedding light on the need for further research.

## Conclusions

In conclusion, autoimmune encephalitis (AE) is a group of immune-related neuropsychiatric disorders characterized by antibodies affecting neuronal structures. Recent research has reshaped the landscape of AE, introducing new syndromes and biomarkers, leading to a better understanding of this complex disorder. Diagnostic criteria emphasize subacute onset symptoms and corroborating abnormalities in brain MRI, cerebrospinal fluid, or EEG patterns. Treatment involves intensive immunotherapy, with varying prognoses linked to AE subtypes and underlying cancer. The case of a 62-year-old woman highlights the diagnostic challenges and therapeutic complexities of AE, from cognitive decline to functional impairment. Imaging studies, antibody detection, and a nuanced treatment course underscore the multidisciplinary approach essential for understanding and managing AE.
